# The impact of pH on proteolytic activity in wound fluid: Implications for acid therapy

**DOI:** 10.1016/j.jbc.2025.110723

**Published:** 2025-09-15

**Authors:** Elany Barbosa da Silva, Meredith J. Crane, Lawrence Liu, Danielle J. Gelsinger, Alexander R.D. Jordon, Phuong Le, Jack G. Haggett, Samuel A. Myers, Robin L. McKinney, Craig P. Eberson, Amanda M. Jamieson, Anthony J. O’Donoghue

**Affiliations:** 1 Skaggs School of Pharmacy and Pharmaceutical Sciences, Center for Discovery and Innovation in Parasitic Diseases, University of California San Diego, La Jolla, California, United States; 2 Department of Molecular Microbiology & Immunology, Brown University, Providence, Rhode Island, United States; 3 Laboratory for Immunochemical Circuits, Division of Signaling and Gene Expression, La Jolla Institute for Immunology, La Jolla, California, United States; 4 Division of Pediatric Critical Care, Hasbro Children’s Hospital, and the Warren Alpert Medical School of Brown University, Providence, Rhode Island, United States; 5 Pediatric Orthopedics Division, Hasbro Children’s Hospital, and the Warren Alpert Medical School of Brown University, Providence, Rhode Island, United States

**Keywords:** proteolytic activity, wound fluid, acid treatment, aspartic acid protease, pepstatin, MSP-MS, fluorogenic substrate

## Abstract

Wound healing necessitates a balance between synthesis and breakdown of extracellular matrix components, which is tightly regulated by proteases and their inhibitors. While studies have demonstrated that citric and acetic acid treatments enhance healing in recalcitrant wounds, the underlying proteolytic mechanisms remain elusive. In this study, we systematically evaluated changes in the proteolytic activity of murine wound fluid upon acidification. A library of 228 synthetic peptides served as reporters of protease activity at pH 7.4, pH 5.0, and pH 3.5. The peptide digestion patterns differed at each pH, revealing that proteases active at pH 7.4 are inactivated at pH 3.5. Notably, cathepsin D emerged as the dominant active enzyme at pH 3.5, and its activity was inhibited by pepstatin. Using a fluorogenic substrate, we quantified cathepsin D activity across varying pH levels and demonstrated optimal activity between pH 3.0 and 3.8. This activity was detectable as early as 1 day postinjury and persisted over the following 10 days. Importantly, human wound fluid exhibited the same activity profile, validating the mouse model as a relevant system for studying acid-mediated wound healing processes.

The successful healing of adult skin involves carefully coordinated phases, including hemostasis, inflammation, proliferation, and remodeling. Disruption of any of these stages can derail the process, leading to chronic nonhealing wounds (1, 2). Elderly individuals and those with diabetes are at especially high risk of developing poorly healing wounds, requiring an estimated $28B annually in health care–associated expenditures (3, 4). With over 20% of the US population expected to be of age 65 or older by the year 2030, and the number of people being diagnosed with type 2 diabetes increasing year after year, the medical and financial costs associated with poorly healing wounds are projected to increase accordingly. Therefore, it is important to better understand the numerous molecular players that coordinate the stages of tissue repair at steady state and in chronic settings to inform new avenues of treatment for poorly healing wounds. Proteases play crucial roles in all the stages of the wound healing process, from the initial hemostasis stage through extracellular matrix remodeling. To date, most of the well-studied proteolytic activities in wound healing are metalloproteinases and serine proteases. Matrix metalloproteinases-2 (MMP-2) and -9 (MMP-9) degrade the components of the extracellular matrix, assist in regenerating injured tissues, and help cellular migration to the wound area (5). Serine proteases, such as cathepsin G, elastase, and urokinase-type plasminogen activator, are expressed by immune cells and endothelial cells in the wound microenvironment, and they are mainly involved in the re-epithelialization process and degradation of growth factors. Of note, each of these enzymes is optimally active at neutral pH (1).

The typical pH range of human skin is between 4.2 and 5.6, which creates an acidic environment that serves as a protective barrier against bacterial overgrowth (6). In contrast, acute wounds exhibit a neutral to alkaline pH spanning from 6.5 to 8.5, and chronic wounds tend to fall within the range of 7.2 to 8.9 (6). Chronic wounds have been associated with increased protease levels that are influenced by the pH milieu of the wound (7).

Reagents that can lower wound pH, such as citric acid (CA) and acetic acid, have been used to promote wound healing. These treatments have been shown to have a positive correlation with accelerated wound healing (8, 9, 10, 11). In a notable case study of a patient with multiple leg ulcers, 33 applications of a 3% CA resulted in complete healing (12). This outcome stands in contrast to the ineffectiveness of conventional therapies, whereby amputation had been recommended to this patient prior to CA treatment. In another case study, a daily application of 3% CA for 11 days achieved resolution of a nonhealing tuberculous sinus. Notably, the sinus had shown resistance to all conventional antimicrobial therapies and local care interventions (8). In a larger study consisting of 115 patients with diabetic foot ulcers infected with diverse bacteria, treatment using a CA gel resulted in 106 patients experiencing healing (13). An acidic environment is generally unfavorable to bacteria, so the treatments are thought to reduce infection. Studies have shown that CA treatment results in a higher percentage of re-epithelialization, a slenderer epithelial layer, improved wound contracture, and elevated levels of collagen deposition in noninfected wounds (14). However, the mechanism of how acidification of wounds affects proteolysis during healing has not been fully elucidated.

Enzymes that are well characterized in the wound healing setting, like MMP-2, MMP-9, and urokinase-type plasminogen activator, are optimally active at neutral pH (15, 16, 17) and therefore are unlikely to be functional in acidic conditions. Therefore, we sought to assess protease activity in an acidified wound. We utilized a peptide digestion assay known as multiplex substrate profiling by mass spectrometry (MSP-MS) to determine the peptide cleavage profile generated by wound fluid proteases at three pH conditions (as illustrated in Fig. 1). More specifically, this assay consists of a library of 228 strategically designed 14-mer peptides. Upon addition of a biofluid sample, cleavage of any of the 2964 peptide bonds (228 peptides × 13 bonds) in the library by proteases can be detected using tandem mass spectrometry (MS). This method has previously been used to discover new protease activities in biological samples, such as cancer cell secretions (18), plasma (19), gastric juice (20), and pancreatic cyst fluid (21). We anticipated that peptides in this library would be cleaved by proteases in the wound fluid when assayed at neutral pH, as these proteases have already been well characterized. We hypothesized that the protease activity in the same wound fluid samples would be reduced when assayed at acidic conditions, since the optimum pH of MMPs and serine proteases is close to neutral pH (15, 16, 17). However, acidification of wound fluid activates aspartic acid proteases, and the total number of peptides that are cleaved is higher at pH 3.5 when compared with pH 7.4. In this study, we performed an in-depth characterization of the enzyme activity derived from aspartic acid proteases in murine wound fluid and then revealed that similar activity exists in human wound fluid. This study sheds light on the changes in protease activity upon acidification of wounds.

**Figure 1:**
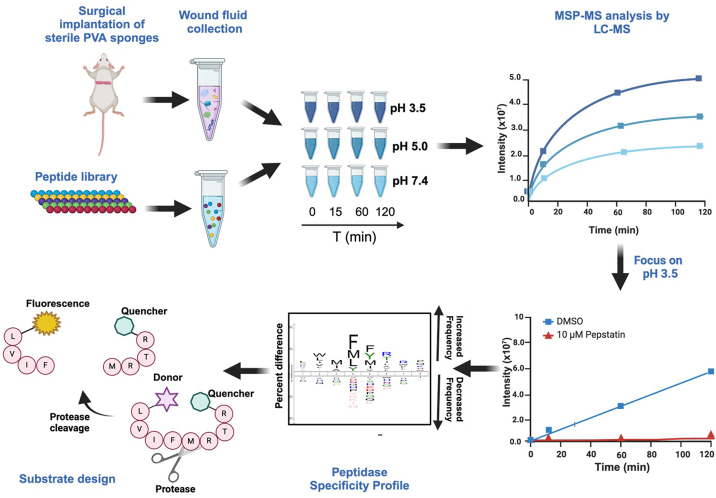
**Overview of the substrate profiling studies with murine wound fluid at pH 3.5, 5.0, and 7.4 and the development of fluorogenic reporter substrates**.

## Results

**Figure 2:**
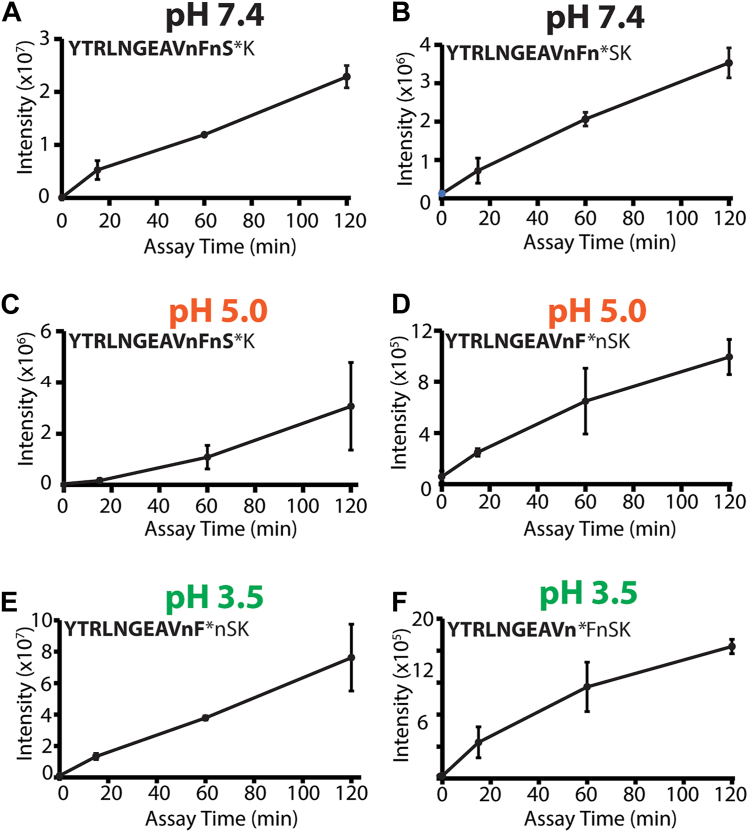
**pH-dependent cleavage of YTRLNGEAVnFnSK by murine wound fluid proteases over time.***A,* peptide was cleaved between S and K at pH 7.4, and the cleavage site is indicated by ∗. The cleavage product (highlighted in bold font) was quantified by LC–MS/MS after 0-, 15-, 60-, and 120-min incubation. *B,* a second cleavage product was detected and quantified at pH 7.4. *C* and *D,* cleavage products quantified at pH 5.0. *E* and *F,* cleavage products quantified at pH 3.5. All assays were performed in four technical replicates using a pooled wound fluid sample from three mice. Lowercase n corresponds to norleucine.

To uncover the protease activities in murine wound fluid at different pH conditions, wound fluid was pooled from three mice 5 days post polyvinyl alcohol (PVA) sponge implantation and incubated with an equimolar mixture of 228 14-mer peptides in buffers that varied by pH. As a control for normal protease activity, the wound fluid was incubated with the peptide mixture in pH 7.4 assay buffer. Assays were also performed in pH 5.5 and pH 3.5 buffers to mimic a mildly acidic and moderately acidic environment, respectively. Following 2 h of incubation with the peptide mixture, cleaved products were found in all pH conditions; however, the site of peptide cleavage often differed (Files S1–S3). Only one peptide in the library, with the sequence YTRLNGEAVnFnSK, was cleaved by proteases at all pH conditions; however, the location of the cleavage site differed at different pH conditions. At pH 7.4, proteases cleaved between nS and SK. However, at pH 5.0, cleavage occurred between Fn and SK, whereas at pH 3.5, cleavage occurred between nF and Fn (Fig. 2). As there is no overlap between the cleavage sites generated at pH 7.4 and pH 3.5, we hypothesize that proteases active at one pH are inactive at the other. In addition, protease activity at pH 5.0 is likely because of some of the neutral and acid proteases retaining activity at this intermediate condition.

**Figure 3:**
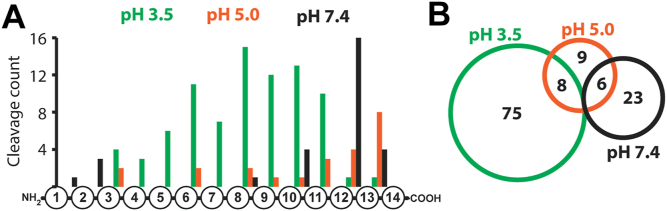
**Quantitative multiplex substrate profiling of murine wound fluid.***A,* distribution of murine wound fluid protease cleavage sites within 14-mer peptides. *B,* the Venn diagram shows the number of cleavage sites shared between the three different pH conditions.

When murine wound fluid was assayed at pH 7.4, proteases cleaved at 29 sites. The distribution of these cleavage sites across the 14-mer peptides, as illustrated in Figure 3*A*, revealed that 16 of the 29 cleavage sites occurred between the 12th and 13th residues. The proteases responsible for cleavage at this site are categorized as dicarboxypeptidases, as these enzymes remove two amino acids from the carboxy terminus. In addition, four peptides were cleaved between the 13th and 14th amino acids, indicating the presence of monocarboxypeptidases, while the remaining cleavage occurred near the N terminus or in the middle of the peptides.

At pH 5.0, the distribution of cleavage sites differed from that seen at pH 7.4 conditions. Here, 8 of the 23 cleavage sites were between the 13th and 14th residues, whereas only four peptides were cleaved between the 12th and 13th amino acids. These data revealed that the monocarboxypeptidase activity is stronger than the dicarboxypeptidase activity at pH 5.0. Finally, at pH 3.5, we were surprised to detect 83 cleaved peptides, generated by proteases in the wound fluid. The distribution of these sites was spread between the 3rd and 12th amino acids, and only one peptide was cleaved between the 12th and 13th residues, and another one between the 13th and 14th residues. This distribution indicates that most proteases that are active at pH 3.5 can be defined as endoproteases, as these enzymes preferentially cleave away from the N and C termini.

We next assigned each of the potential 2964 cleavage sites (228 peptides × 13 peptide bonds) within the library to a unique identifier and then determined which of them were cleaved by wound fluid proteases across the three pH conditions. We found that there was no overlap between the sites cleaved by proteases at pH 3.5 and pH 7.4, whereas there were eight sites in common between pH 3.5 and pH 5.0 and six sites in common between pH 5.0 and pH 7.4 (Fig. 3*B*). In addition, there were proteases that were active at pH 5.0 and not at the more acidic and neutral conditions, since there were nine cleavage sites that were generated only at pH 5.0. These data reveal that acidification of wound fluid results in the activation of distinct acid-acting proteases and inactivation of the neutral-acting proteases.

**Figure 4:**
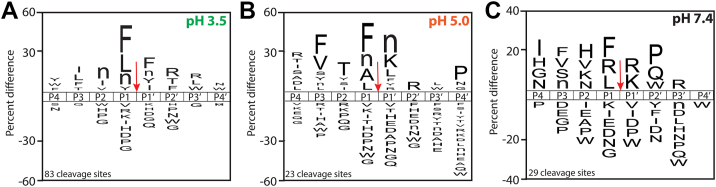
**iceLogo plots illustrating the cleavage profiles.** Murine wound fluid samples taken 5 days after injury were assayed at *A,* pH 3.5, *B,* 5.0, and *C,* 7.4. Data represent a pooled wound sample from three mice assayed in quadruplicate reactions. The *red arrow* indicates the site of cleavage. iceLogo plots the relative frequencies of amino acid residues at each of the P4 to P4′ positions of the cleaved peptides, where the height of each amino acids represents “percent difference,” defined as the difference in frequency for an amino acid appearing in the “experimental dataset” relative to the “reference dataset.” Ranked preferred amino acids are shown above the midline, whereas unpreferred amino acids are represented below the midline.

We next generated a substrate specificity profile at each pH condition to illustrate the similarities and differences in the murine protease activity with changing pH (File S4). At pH 3.5, peptides were cleaved frequently when Phe, Leu, norleucine (Nle), and Tyr were in the P1 position, whereas Phe, Nle, Tyr, and Ile were favored at P1′. At other positions, Arg at P2′ and P3′ was frequently observed, whereas nonpolar amino acids were found with the highest frequency at P2 and P3 (Fig. 4*A*). At pH 5.0, nonpolar amino acids were generally found at P3, P1, and P1′, whereas Pro was favored at P4′ and Lys and Arg were favored at P1′ and P2, respectively (Fig. 4*B*). At pH 7.4, positively charged amino acids, such as Arg and Lys, were favored at P1′, whereas nonpolar amino acids in addition to Arg were also frequently found at P1 (Fig. 4*C*). These findings underscore the diverse protease activities present in wound fluids across a broad pH range.

**Figure 5:**
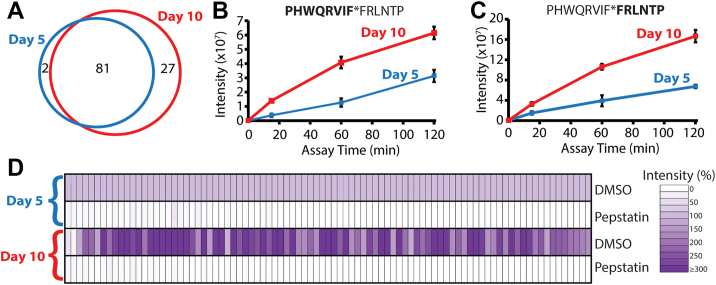
**Comparation of proteolytic activity in murine wound fluid by MSP-MS, 5 and 10 days postinjury.***A,* number of cleavage sites detected at pH 3.5 in wound fluid 5 and 10 days postinjury. *B* and *C,* quantification of the time-dependent accumulation of select N-terminal and C-terminal fragments. *D,* normalized intensity of proteolytic cleavage products in the presence and absence of pepstatin. Each *rectangle* represents the average intensity of a cleaved peptide product (n = 4 technical replicates) normalized to the intensity of the same cleaved peptide that was quantified in the day 5 sample. The *lighter color* indicates that the cleaved peptide decreased in intensity, whereas the *darker color* indicates that the peptide increased. MSP-MS, multiplex substrate profiling by mass spectrometry.

Following these studies at different pH conditions, we then focused our attention on the protease activities present at pH 3.5 since these enzymes are likely to be active in acid-treated wounds. We first sought to determine if differences in protease activity exist in murine wound fluid taken 5 days and 10 days after injury. Wound fluid from day 10 after injury was subjected to MSP-MS analysis in pH 3.5 assay buffer (File S5). In total, 108 cleavage sites were quantified, of which 81 were identical to those found in the day 5 sample (Fig. 5*A*). Most of the additional 27 cleavage sites detected on day 10 were also observed in the day 5 sample; however, the cleavage products were not sufficiently abundant to pass our stringent statistical testing. These data reveal that the same acid-acting proteases exist in wound fluid on day 5 and day 10, but they are likely to be present in higher concentrations on day 10. To evaluate further, the cleavage products generated in each dataset were directly compared. One of the most efficiently cleaved substrates was PHWQRVIFFRLNTP, where cleavage occurs between FF (phenylalanine) residues. We quantified the time-dependent accumulation of both the N-terminal fragment, PHWQRVIF (Fig. 5*B*), and the C-terminal fragment, FRLNTP (Fig. 5*C*), by proteases present in wound fluid from day 5 and 10 postinjury. In both assays, the products were generated more rapidly by proteases in the day 10 sample.

To compare all cleavage sites, we first normalized the intensities of each cleavage product found after 120 min of incubation in the day 5 wound fluid sample and then quantified the relative abundance of the same cleavage product generated by proteases in the day 10 sample. We show that almost all peptide products were higher in intensity following incubation with day 10 wound fluid, with some being more than three times higher (Fig. 5*D*).

We preincubated the wound fluid in this assay with the broad-spectrum aspartic acid protease inhibitor, pepstatin. This inhibitor was chosen as aspartic acid proteases are generally most active at acidic pH, and therefore, these enzymes were likely to dominate in the pH 3.5 assay conditions. In addition, our previous work with aspartic acid proteases in bovine secretory vesicles revealed that aspartic acid proteases had endoprotease activity and a preference for cleaving the peptide library between Phe–Phe residues (22), as is also found in Figure 4*A*. In the presence of pepstatin, the intensity of all cleavage products was reduced and was often not detected above background (Fig. 5*D*). These studies reveal that aspartic acid proteases are the dominant enzyme family in acidified wound fluid.

While the MS-based studies allowed us to discover many cleaved peptides, we sought to identify a fluorogenic substrate that could report on the aspartic acid protease activity in a microplate assay format. Previous studies identified a reporter substrate for cathepsin D and E that contains the sequence GKPILFFRL, flanked on the N terminus by 7-methoxycoumarin-4-acetyl (MCA) and on the C terminus by the quencher 2,4-dinitrophenol-lysine (22). This substrate contains the sequence ILFFRL, which closely matches the P3 to P3′ substrate profile of wound fluid proteases at pH 3.5 (Fig. 4*A*).

**Figure 6:**
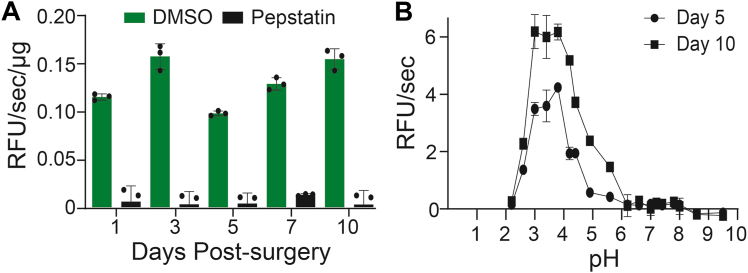
**Murine wound fluid protease activity.***A,* protease activity in acidified wound fluid (pH 3.5) from day 1 to 10 in the absence and presence of pepstatin. *B,* superposition of pH curves for murine wound fluid from day 5 and 10 postinjury using GKPILFFRL substrate. Each data point corresponds to technical triplicates from one pooled wound fluid sample derived from three mice.

When murine wound fluid collected on days 1, 3, 5, 7, and 10 was incubated with the GKPILFFRL substrate, the specific activity at pH 3.5 ranged from 0.1 to 0.16 relative fluorescence units per second per microgram of protein (RFU/s/μg). In all samples, this protease activity was reduced by 90% or more in the presence of pepstatin (Fig. 6*A*), thereby confirming that the fluorogenic substrate is reporting on an aspartic acid protease. In addition, using this substrate, we showed that the specific activity was higher for the day 10 sample relative to day 5 wound fluid samples, as was seen with the MSP-MS assay.

We next assayed wound fluid from days 5 and 10 postinjury across a wide pH range to determine the optimal environment in which the aspartic acid proteases retain catalytic activity. Activity was low at pH 2.2 but increased steadily as the pH increased to 3.8. The maximum activity was found to be between pH 3.0 and pH 3.8. Above pH 3.8, activity decreased, and no fluorescence change above the background was detected at pH 6.2 and above (Fig. 6*B*). These data reveal that the aspartic acid protease present in wound fluid is optimally active between pH 3.0 and 3.8 but is inactive at neutral pH. Therefore, these enzymes cannot contribute to wound proteolysis at neutral pH but are likely to be the dominant activity in acidified wounds.

To identify the aspartic acid protease, wound fluid from three mice was subjected to proteomic analysis, and a total of 735 distinct proteins were found, of which 47 were categorized as proteases (File S6). When these enzymes were classified into different families based on their catalytic residues, only one aspartic protease, cathepsin D, was present (Table 1). These data confirm that cathepsin D is the major aspartic acid protease in murine wound fluid.

**Table 1:** Proteomic analysis of murine wound fluid identified 47 proteases that can be categorized into one of five families

UniProt ID	Metalloprotease	UniProt ID	Serine protease
P33434	72 kDa type IV collagenase (MMP-2)	Q91X79	Chymotrypsin-like elastase family member 1
Q8VCT3	Aminopeptidase B	P16294	Coagulation factor IX
P09470	Angiotensin-converting enzyme	O88947	Coagulation factor X
P98063	Bone morphogenetic protein 1	Q80YC5	Coagulation factor XII
Q9JHH6	Carboxypeptidase B2	Q8CG16	Complement C1r-A subcomponent
Q9JJN5	Carboxypeptidase N catalytic chain	Q8CG14	Complement C1s-1 subcomponent
Q9WVJ3	Carboxypeptidase Q	P21180	Complement C2
Q9CPY7	Cytosol aminopeptidase	P04186	Complement factor B
Q9D1A2	Cytosolic nonspecific dipeptidase	P03953	Complement factor D
Q8C255	Dipeptidase 2	Q61129	Complement factor I
Q99KK7	Dipeptidyl peptidase 3	Q91WP0	Mannan-binding lectin serine protease 2
P24527	Leukotriene A-4 hydrolase	Q61096	Myeloblastin
P34960	Macrophage metalloelastase (MMP-12)	Q3UP87	Neutrophil elastase
P41245	Matrix metalloproteinase-9 (MMP-9)	P26262	Plasma kallikrein
O70138	Neutrophil collagenase (MMP-8)	P20918	Plasminogen
Q11011	Puromycin-sensitive aminopeptidase	Q9QUR6	Prolyl endopeptidase
P28862	Stromelysin-1 (MMP-3)	P19221	Prothrombin
Q11136	Xaa-Pro dipeptidase	P33587	Vitamin K-dependent protein C
			
	Cysteine protease		Threonine protease
Q8R016	Bleomycin hydrolase	O35955	Proteasome subunit beta type-10
O35350	Calpain-1 catalytic subunit	P28063	Proteasome subunit beta type-8
O08529	Calpain-2 catalytic subunit	P28076	Proteasome subunit beta type-9
P10605	Cathepsin B		
O70370	Cathepsin S		Aspartic acid protease
Q9WUU7	Cathepsin Z	P18242	Cathepsin D
O89017	Legumain		

**Figure 7:**
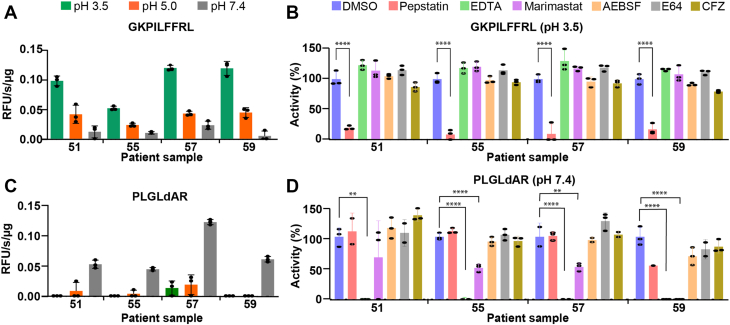
**Human wound fluid protease activity.***A,* activity profile of wound fluid proteases using the GKPILFFRL substrate when assayed across different pH conditions. *B,* inhibition profile of acid protease. Assays were performed in the absence and presence of 10 μM pepstatin, 1 mM EDTA, 100 μM marimastat, 1 mM AEBSF, 10 μM E64, and 10 μM CFZ inhibitors. *C,* activity profile of wound fluid proteases using the PLGLdAR substrate when assayed across different pH conditions. *D,* inhibition profile of MMP-2 protease in wound fluid. Each bar corresponds to one assay performed in triplicate wells. ∗∗*p* < 0.01; ∗∗∗∗*p* < 0.0001 using one-way ANOVA followed by Dunnett’s multiple comparisons test *versus* control. AEBSF, 4-(2-aminoethyl)benzenesulfonyl fluoride hydrochloride; CFZ, carfilzomib; E64, trans-epoxysuccinyl-l-leucylamido(4-guanidino)butane; MMP-2, matrix metalloproteinase 2.

To determine if cathepsin D activity detected in murine wound fluid is equivalent in human samples, we obtained draining wound fluid from one adolescent idiopathic scoliosis (AIS) patient (#55) and three neuromuscular scoliosis (NMS) patients (#51, #57, and #59) who had undergone spinal fusion surgery. Encouragingly, the cathepsin D substrate (GKPILFFRL) was also cleaved by proteases in the patient samples (Fig. 7*A*). Activity was highest at pH 3.5 and decreased as the pH increased to pH 5.0 and pH 7.4. Using a panel of class-specific protease inhibitors, we showed that activity at pH 3.5 was inhibited only by the aspartic acid protease inhibitor, pepstatin, and not by EDTA (metalloprotease inhibitor), marimastat (MMP inhibitor), 4-(2-aminoethyl)benzenesulfonyl fluoride hydrochloride (AEBSF) (serine protease inhibitor), *trans*-epoxysuccinyl-l-leucylamido(4-guanidino)butane (E64) (cysteine protease inhibitor), or carfilzomib (proteasome inhibitor). These data reveal that cathepsin D is also active in human wounds upon acid treatment.

As a control, we also tested a human MMP-2 substrate (PLGLdAR) (23) in the same three pH conditions, and it was found to be cleaved exclusively at pH 7.4 for patient samples 51, 55, and 59 (Fig. 7*C*). For patient 57, cleavage of this substrate was also detectable at pH 5.5 and pH 3.5, albeit at a lower level than at pH 7.4. Using the same set of inhibitors, we show that cleavage of PLGLdAR is not inhibited by pepstatin but is inhibited by EDTA and marimastat, strongly suggesting that activity is due to MMP-2 (Fig. 7*D*).

**Figure 8:**
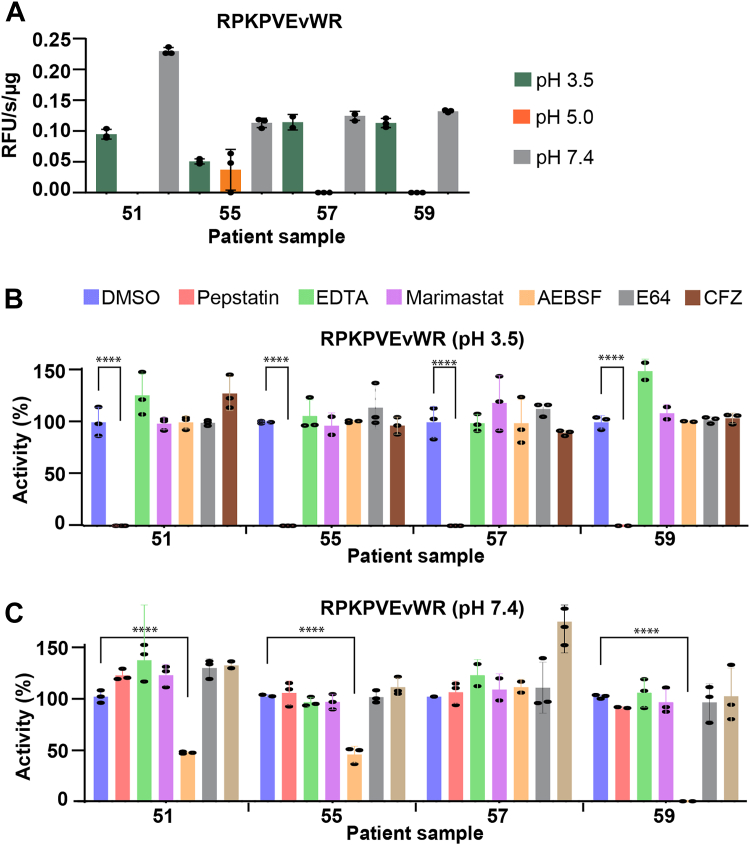
**Human wound fluid protease activity.***A,* protease activity assays of human wound fluid proteases using RPKPVEvWR as substrate when assayed across different pH conditions. *B,* inhibition profile of acid protease. Assays were performed in the absence and presence of 10 μM pepstatin, 1 mM EDTA, 100 μM marimastat, 1 mM AEBSF, 10 μM E64, and 10 μM CFZ inhibitors at pH 3.5. *C,* inhibition profile of serine proteases in wound fluid at pH 7.4. Each *bar* corresponds to one assay performed in triplicate wells. ∗∗∗∗*p* < 0.0001 using one-way ANOVA followed by Dunnett’s multiple comparisons test *versus* control. AEBSF, 4-(2-aminoethyl)benzenesulfonyl fluoride hydrochloride; CFZ, carfilzomib; E64, trans-epoxysuccinyl-l-leucylamido(4-guanidino)butane.

The two fluorogenic substrates, GKPILFFRL and PLGLdAR, are each selective for proteases that are active at acid or neutral pH, respectively. We sought to find a reporter substrate that is cleaved at both pH conditions to directly compare the cleavage rate. We screened a small library of in-house fluorescent peptides and determined that RPKPVEvWR was a substrate that could be cleaved by human wound fluid proteases at pH 3.5 and pH 7.4 (Fig. 8*A*). This substrate was originally developed for cleavage by MMP-3 (24). For patients 51, 57, and 59, no cleavage of this substrate occurred at pH 5.0, indicating that the enzyme(s) responsible for cleaving this substrate at pH 7.4 are not active at pH 5.0. When assayed at pH 3.5, this substrate was cleaved by all wound fluid samples, and this activity was inhibited by pepstatin, revealing that it was cleaved by cathepsin D (Fig. 8*B*). When assayed at pH 7.4, AEBSF significantly reduced activity in three of four patients, revealing that a serine protease was responsible for this enzyme activity. No reduction was seen in the presence of the MMP inhibitors, EDTA and marimastat (Fig. 8*C*), making it likely that MMP-3 is not active in these samples. Overall, this substrate is hydrolyzed equally by serine proteases at neutral pH and cathepsin D at pH 3.5.

These studies show that pH plays a key role in determining the active proteases in wound fluid in both animal models and human wound fluid samples and suggest that we can potentially improve wound healing rate and quality by carefully adjusting the pH of the wound using CA, acetic acid, or other acidic reagents.

## Discussion

Our study revealed the presence of cathepsin D in wound fluid that is active only under acidic pH conditions. This finding is important as it sheds light on the proteolysis that occurs when wounds are treated with CA and acetic acid. Achieving a balance between the synthesis and breakdown of matrix components at neutral pH is crucial for wound healing. This balance requires the precise regulation of key proteases, such as cathepsin G, elastase, plasmin, and MMP2, and protease inhibitors, such as serpins and tissue inhibitors of metalloproteinases (1, 5). Greener *et al*. (15) demonstrated that these enzymes are optimally active from pH 7 to 9, which aligns with the pH range typically found in wounds. Elevated protease activity is associated with poor wound healing, which has led to the development of numerous wound dressings designed to reduce protease activity through inhibition or absorption (25, 26). Acidification of wounds to pH 4 results in an 80% reduction in the activity of these neutral-acting enzymes (15), but our study reveals that cathepsin D and other proteases become active in these conditions.

The pH of the skin surface is typically between pH 4 and 6, and this acidity is crucial for maintaining a healthy stratum corneum and for epidermal barrier homeostasis (27). Interestingly, the pH of skin is increased in the elderly population, causing increased serine protease activity and resulting in premature degradation of corneodesmosomes and increased desquamation (28, 29). Cathepsins L, B, and D are all acid-acting proteases that are present in the epidermis, and Western blotting studies revealed that they were mostly present as inactive proenzymes (30). While previous proteomic studies found these enzymes in wound fluid (31), our own mouse wound fluid studies specifically identified cathepsins B and D but not cathepsin L (Table 1). However, we did find cathepsin S, an acid-active homolog of cathepsin L, in the wound fluid. Therefore, it is likely that the acidification of wound fluid activates all these enzymes.

When murine wound fluid was acidified to pH 3.5, we detected robust cleavage of a diverse peptide library and a resulting substrate preference for cleavage between hydrophobic amino acids. We have previously observed a similar profile when characterizing human cathepsin D using the same peptide library (22). Addition of pepstatin, a pan–aspartic acid inhibitor, eliminated this activity, revealing that the enzyme responsible for this activity was likely to be cathepsin D. This observation was further supported by the efficient cleavage of a cathepsin D fluorogenic substrate in wound fluid following acidification to pH 3.5 and the discovery of this enzyme as the only aspartic acid protease in wound fluid. Taken together, these data reveal that cathepsin D in wound fluid is activated upon exposure to acid pH.

The protease activity detected in wound fluid at pH 5.0 may be partly because of cathepsin D, as this retains some activity up to pH 5.5. However, additional proteases are active at pH 5.0, which could be cathepsin B and S, as these enzymes are optimally active from pH 4.5 to pH 6.0. Both enzymes are inactivated by the cysteine protease inhibitor, E64, and therefore, future studies will be performed to determine if this inhibitor can uncover the role of cysteine proteases in mildly acidic conditions.

CA treatment is known to promote wound healing through several mechanisms, including stimulating epithelialization *via* fibroblast proliferation, increasing local oxygen levels, and inhibiting microbial growth and virulence (10). Prolonged wound surface acidification has also been associated with improved healing, likely because of increased cellular oxygen availability within the wound (32). In addition, a lower wound pH correlates with increased DNA synthesis (33), suggesting a mechanism for accelerated tissue repair. This study now identifies a novel potential mechanism by which CA may facilitate wound healing: by promoting the activity of cathepsin D, while simultaneously inhibiting the activity of serine and metalloproteases.

In this study, we have revealed that cathepsin D is the predominant protease in acid-treated wounds. Interestingly, this enzyme is not enzymatically active above pH 5.5, suggesting that it does not play a functional role in wound healing under normal pH conditions. This discovery opens up several crucial questions for future research. Is cathepsin D merely a "bystander" enzyme that is activated during acid treatment, or is it a functionally relevant enzyme that actively contributes to the wound healing process? If it does play a healing role, then understanding the optimal duration of acid treatment and the best type and concentration of acid to promote its activity becomes paramount. Ultimately, before the widespread clinical use of acid in wound healing, we need a much deeper understanding of the biochemical mechanisms involved and a rigorous evaluation of safety. Our current findings lay the groundwork for a more targeted approach, allowing us to specifically influence protease activities during wound healing.

## Experimental procedures

### Mouse models and wound fluid retrieval

All animal studies were approved by the Brown University Institutional Animal Care and Use Committee (protocol #22-04-0001) and performed in accordance with the Guide for the Care and Use of Animals of the National Institutes of Health. C57BL/6J mice were bred in-house. Male mice aged 8 to 12 weeks were used for studies. Wound fluid samples were collected using the subcutaneous PVA sponge implantation model, which allows for the retrieval of wound fluid with minimal manipulation (34, 35, 36). The model recapitulates the phases of acute wound healing, including inflammatory, angiogenic, and fibrotic responses seen in soft tissue wounds, for up to 2 weeks following implantation. For PVA sponge implantation surgery, mice were placed under anesthesia and analgesia using ketamine (80–100 mg/kg) and xylazine (8–10 mg/kg), respectively. The dorsum was shaved and cleaned with povidone–iodine solution and 70% ethanol. Under sterile conditions, a 2 cm full-thickness incision was made along the dorsal midline. Six sterile 1 cm × 1 cm × 0.5 cm PVA sponges (PVA unlimited) were implanted into subcutaneous pockets on either side of the midline. The incision was then closed using stainless steel surgical clips. In this model, fluid that accumulates in response to the injury is captured by the sponges, which were then removed from the mice at various time points (day 1, 3, 5, 7, and 10). To isolate wound fluid, PVA sponges were placed into the barrel of a 5 ml syringe that was nested in a 15 ml culture tube. The tube was centrifuged at 500*g* for 10 min, allowing fluid to be drawn out of the sponges into the collection tube. Cell-free fluid was stored at −80 °C (37).

### Spinal fusion surgical wound drainage sample collection

The study using human samples was conducted in accordance with the Declaration of Helsinki principles and approved by the Lifespan Institutional Review Board (IRB #1135267). Informed consent was obtained from the patient or the guardian. Drainage samples were collected from AIS or NMS patients recovering from spinal fusion surgery. As part of the standard protocol for pediatric spinal fusions, drains were placed either subcutaneously for AIS patients or in the submuscular space for patients with NMS, the latter group usually undergoing a multilayered closure with muscle flaps because of well-documented healing issues in this group. While drains for the AIS patients were usually removed by the second or third postoperative day, the drains for the NMS patients usually had higher output because of their location below the muscle layer and thus remained in place for the entire hospitalization in most cases, resulting in longer sampling time. Daily samples were collected from the effluent of wound drains placed at the surgical incision. Once collected, samples were kept on ice until the time of processing. Drainage fluid was centrifuged at 300*g*, and the cell-free supernatant was stored at −80 °C.

### Bicinchoninic acid assays

Protein in murine and human wound fluid samples was quantified with the Bicinchoninic Acid Protein Assay kit (ThermoFisher Pierce) using the 96-well plate protocol recommended by the manufacturer. A serial dilution of bovine serum albumin standards was evaluated in parallel with a one in 10 and one in 50 dilution of each wound fluid sample. The standard (25 μl) or unknown was then incubated with 200 μl of the working reagent, which was included in the assay kit, at 37°C for 30 min. The plate was then read at 562 nm using a Synergy HTX (Biotek) plate reader. A standard curve was created, and the dilution of each sample that fell within this range was computed and used to determine the total protein concentration in the sample. These stock concentrations were then used to normalize the activity to protein concentration.

### Protease cleavage profile by MSP-MS

MSP-MS utilizes a library of 228 tetradecapeptides designed to contain all neighbor and near-neighbor cleavage sites (38). These peptides contain 18 of the 20 natural amino acids but lack cysteine and methionine because of their propensities to form disulfide bonds and become oxidized, respectively. The oxidation-resistant norleucine (indicated as Nle or lowercase n) was included as a substitute for methionine. Wound fluids were extracted from three mice on day 5 postinjury. They were diluted 110-fold in each of the following assay buffers: assay buffer 1: 20 mM sodium citrate (pH 3.5), 100 mM sodium chloride (NaCl); assay buffer 2: 20 mM sodium acetate (pH 5.0) and 100 mM NaCl; and assay buffer 3: Dulbecco’s PBS pH 7.4 (Gibco). In parallel, the library of peptides was diluted 21.9-fold in the same three buffers such that the concentration of each peptide was 1 μM. Then, an equal volume of wound fluid in assay buffer was mixed with peptides in the same assay buffer such that the final concentration of each peptide was 0.5 μM. Assays were incubated at 25 °C for 15, 60, and 120 min. At each time point, 20 μl of the reaction mixture was removed and quenched by the addition of urea (Sigma–Aldrich) to a final concentration of 4 M. Samples were immediately stored at −80 °C. Control reactions consisted of murine wound fluid samples preincubated with 4 M urea to inactivate the enzymes prior to the addition of the peptide library. All samples were desalted using a C18 column and dried in a vacuum centrifuge as outlined previously (39). All assays were performed in four technical replicates.

In a follow-up study, murine wound fluid from days 5 and 10 was diluted 110-fold in assay buffer 1 containing 20 μM of pepstatin or 0.2% dimethyl sulfoxide (DMSO) as the vehicle control. The reaction was incubated for 60 min and then was mixed with an equal volume of the peptide library diluted in assay buffer 1. Assays were incubated at 25 °C for 15, 60, and 120 min, and 20 μl of the reaction mixture was removed and quenched by the addition of urea (Sigma–Aldrich). The control reaction (0 min) consisted of wound fluid preincubated with 4 M urea prior to the addition of the peptide library.

For each MSP-MS assay sample, ∼0.4 μg of peptides was injected into a Q-Exactive Mass Spectrometer (Thermo Fisher Scientific) equipped with an Ultimate 3000 HPLC (Thermo Fisher Scientific). Peptides were separated by reverse-phase chromatography on a C18 column (1.7 μm bead size, 75 μm × 25 cm, 65 °C) at a flow rate of 300 nl/min using a 60-min linear gradient from 5 to 30% B, with solvent A: 0.1% formic acid (FA) in water and solvent B: 0.1% FA in acetonitrile. Survey scans were recorded over a 150 to 2000 *m/z* range (70,000 resolutions at 200 *m/z*, automatic gain control (AGC) target 3 × 10^6^, 100 ms maximum). MS/MS was performed in a data-dependent acquisition mode with higher energy collisional dissociation (HCD) fragmentation (28 normalized collision energy) on the 12 most intense precursor ions (17,500 resolutions at 200 *m/z*, AGC target 1 × 10^5^, 50 ms maximum, dynamic exclusion 20 s). Data were processed using PEAKS 8.5 (Bioinformatics Solutions, Inc). MS^2^ data were searched against the tetradecapeptide library sequences with decoy sequences in reverse order. A precursor tolerance of 20 ppm and 0.01 Da for MS^2^ fragments was defined. No protease digestion was specified. Data were filtered to a 1% peptide-level false discovery rate with the target-decoy strategy. Peptides were quantified with label-free quantification, and data were normalized by median and filtered by 0.3 peptide quality. Missing and zero values were imputed with random normally distributed numbers in the range of the average of the smallest 5% of the data ± SD. Cleaved peptide products at the 15, 60, or 120 min time points were defined as having intensity scores of eightfold or more over peptide products in the 0 min time point with *p* < 0.05 by the two-tailed homoscedastic *t* test.

### Cleavage site analysis by iceLogo

Evaluation of the frequencies of the P4 to P4′ amino acids adjacent to the cleavage sites was conducted using the iceLogo software, version 1.3.8, where the “experimental dataset” consists of the detected cleavage sites defined earlier and the “reference dataset” consists of all possible cleavages within the MSP-MS library of 228 14-mer peptides. *z*-Scores were calculated by the equation (X – μ)/σ, where X is the frequency of the amino acid occurring in the “experimental dataset” at a specific position relative to the cleavage site, μ is the frequency of the amino acid at a specific position in the “reference dataset,” and σ is the SD. *z*-Scores were utilized to generate iceLogo illustrations of the relative frequencies of amino acid residues at each of the P4 to P4′ positions of the cleaved peptides, where heights of the single-letter amino acids represent “percent difference,” defined as the difference in the frequency for an amino acid appearing in the “experimental dataset” relative to the “reference dataset.” Amino acids shown above the midline have positive *z*-scores, indicating preferred amino acids, and amino acids shown below the midline have negative *z*-scores, indicating nonpreferred amino acids.

### Aspartyl protease activity and inhibition assays

Activity of murine wound fluid samples pooled from three mice from day 1, 3, 5, 7, and 10 postinjury was measured by monitoring the cleavage of the fluorescent substrate, MCA-Gly-Lys-Pro-Ile-Leu-Phe-Phe-Arg-Leu-Lys(DNP)-dArg-NH_2_ (shortened to GKPILFFRL) (CPC Scientific) using a Synergy HTX (Biotek) fluorimeter. A 2 mM stock solution of this substrate was prepared in DMSO. All assays were performed in black flat-bottom 384-well plates (Greiner) in a total volume of 30 μl. The final assay consisted of a 1 in 110 dilution of wound fluid in buffer 1 supplemented with 2 mM DTT, 0.01% Tween-20, and 20 μM GKPILFFRLK. Prior to the initiation of the assay, the wound fluid was preincubated with 20 μM pepstatin or 0.2% DMSO for 1 h. In the assay, the change in fluorescence was recorded for 2 h, and enzymatic activity in RFU/s was calculated from the initial rates of the reaction. All assays were run in triplicate wells. Using the total protein content in the assay, the initial rates were then normalized to RFU/s per μg of protein (RFU/s/μg) to allow for comparison across samples.

Activity of human surgical wound fluid samples from one AIS and three NM patients was measured by monitoring the cleavage of the fluorescent substrates, GKPILFFRL, MCA-Arg-Pro-Lys-Pro-Val-Glu-Nva-Trp-Arg-Lys(DNP)-DArg-NH_2_ (shortened to RPKPVEvWR) (CPC Scientific), and MCA-Pro-Leu-Gly-Leu-Dap(DNP)-Ala-Arg-NH_2_ (shortened to PLGLdAR) (CPC Scientific), using a Synergy HTX (Biotek) fluorimeter. A 2 mM stock solution of the substrates was prepared in DMSO. All assays were performed in black flat-bottom 384-well plates (Greiner), in a total volume of 30 μl using three different pH buffers: (1) assay buffer 1, supplemented with 2 mM DTT, 0.01% Tween; (2) assay buffer 2, supplemented with 2 mM DTT, 0.01% Tween; and (3) Dulbecco`s PBS pH 7.4 (Gibco). All substrates were tested at 20 μM using a 1 in 110 dilution of each wound fluid. For inhibitor studies, wound fluid was preincubated for 1 h with 20 μM pepstatin, 1 mM EDTA, 100 μM marimastat, 10 μΜ Ε64, 1 mΜ AΕBSF, and 10 μM carfilzomib. Following the substrate addition, the change in fluorescence was recorded for 2 h. Enzymatic activity in RFU/s was calculated from the initial rates of the reaction. All assays were run in triplicate technical replicates, and DMSO was used as the vehicle control. Using the total protein content in the assay, the results were then normalized to RFU/s/μg to allow for comparison across samples, where necessary.

### Protease activity assays across a pH range

Murine wound fluid samples from days 5 and 10 were each pooled from three mice and assayed with 20 μM GKPILFFRL in 16 different citrate phosphate buffers ranging from pH 2.2 to pH 8.0 and two Tris buffers at pH 8.6 and 9.5. All buffers consisted of 100 mM citrate phosphate or 100 mM Tris–HCl. Experiments were performed in triplicate wells using a Synergy HTX (Biotek) fluorimeter.

### Proteomic studies

Wound fluid was isolated from three mice on day 7 post PVA sponge implantation, and the protein was quantified using a bicinchoninic acid assay. Each sample (50 μg) was reduced and alkylated with DTT and iodoacetamide, respectively, and then digested with one mAU of LysC (Wako; catalog no.: 129-02541) in 8 M urea buffer (8 M urea, 50 mM Tris [pH 8.0], 1 mM EDTA, 1× Halt Protease and Phosphatase Inhibitor Cocktail [Pierce; catalog no.: 78442]) for 2 h at 25 °C, followed by digestion with 1 μg sequencing-grade trypsin (Promega; catalog no.: V5113) overnight at 37 °C. Trypsin was quenched by adding FA to a final concentration of 1%, then the samples were desalted on STAGE tips prepared from C18 resin (Empore; catalog no.: 2215). The concentration of the eluents was determined by nanodrop, then the samples were dried to completion using vacuum centrifugation and resuspended at a concentration of 1 μg/μl in 5% MeCN, 0.1% FA in HPLC-grade water. Total peptide (1 μg) was injected on an Orbitrap Eclipse (Thermo Scientific) instrument equipped with an Easy nLC 1200 (Thermo Scientific) at a flow rate of 200 nl/min on an Aurora Ultimate 25 cm × 75 μm ID, 1.7 μm C18 column (IonOpticks). A 110-min gradient starting from 6% MeCN/0.1% FA to 36% MeCN/0.1% FA for 85 min, followed by 72% MeCN/0.1% FA for 9 min, 90% MeCN/0.1% FA for 6 min, and 60% MeCN/0.1% FA for 10 min was used. Data were acquired using a data-dependent acquisition method. After the survey scan of *m/z* 350 to 2000 was measured in the Orbitrap at 60,000 resolution, the top 20 multiply charged ions were selected for HCD fragmentation. MS/MS spectra were collected in the Orbitrap at a resolution of 15,000. AGC for MS/MS was set to standard with a maximum injection time of 50 ms. Normalized collision energy for HCD was set at 30%. Dynamic exclusion was set to 45 s. Data were searched using Proteome Discoverer against a SwissProt mouse protein database (February 5, 2025 version). Spectra were filtered for precursor masses of 350 to 5000 Da with a precursor mass tolerance of 10 ppm, a fragment mass tolerance of 0.02 Da, and a maximum of two missed cleavages. Methionine oxidation and protein N-terminal acetylation were defined as dynamic modifications. Carbamidomethylation was defined as a static modification. Spectra were correlated with peptides using the Sequest search algorithm. Peptide spectral matches (PSMs) were validated with the Fixed Value PSM Validator node with a maximum delta Cn of 0.05. PSMs were validated based on a strict false discovery rate target of 0.01 and a relaxed false discovery rate target of 0.05.

## Data availability

MS data are available at the following links: ftp://massive.ucsd.edu/v09/MSV000097157/, ftp://massive.ucsd.edu/v09/MSV000097153/, ftp://massive.ucsd.edu/v09/MSV000097154/, and ftp://massive.ucsd.edu/v09/MSV000098877/.

## Supporting information

This article contains supporting information. Excel files containing processed MSP-MS data are used for Figs. S3–S5. The Excel file containing proteomic data is used for Table S1.

## Conflict of interest

The authors declare that they have no conflicts of interest with the contents of this article.

## Supporting information

Supporting File 6

MSP-MS data Day5 pH3.5 Figure 3

MSP-MS data Day5 pH5.0 Figure 3

MSP-MS data Day5 pH7.4 Figure 3

MSP-MS data for Figure 4

MSP-MS data for Figure 5
